# Is personality associated with the lived experience of the NHS England low calorie diet programme: A pilot study

**DOI:** 10.1111/cob.70003

**Published:** 2025-02-25

**Authors:** Stuart W. Flint, Ezra Goldberg, Mohammad Kaykanloo, Stuart Sherman, Duncan Radley, Sarah R. Kingsbury, Louisa Ells

**Affiliations:** ^1^ School of Psychology University of Leeds Leeds UK; ^2^ Scaled Insights, Nexus University of Leeds Leeds UK; ^3^ Obesity Institute Leeds Beckett University Leeds UK; ^4^ Leeds Institute of Rheumatic and Musculoskeletal Medicine & NIHR Leeds Biomedical Research Centre University of Leeds Leeds UK

**Keywords:** artificial intelligence, low calorie diet, type 2 diabetes, obesity

## Abstract

This pilot study explored the use of a novel behavioural artificial intelligence (AI) tool to examine whether personality is associated with the lived experience of the NHS England launched a low calorie diet (LCD). A cross‐sectional survey was disseminated to service users to gather data on emotional wellbeing, physical activity, pain, motivation to manage diabetes, motivation to lose weight, rating of total diet replacement (TDR) products and frequency of using fibre supplements. The scaled insights behavioural AI tool was used to infer personality traits from service users' language construction, and in doing so, examine associations with the outcomes indicated above. Findings show that service users can be profiled by personality, and this can provide a method of understanding programme outcomes. Three clusters of personality traits were identified. Despite this, there was no association between personality features and emotional wellbeing, physical activity, pain, motivation to manage diabetes, motivation to lose weight, rating of TDR products and frequency of using fibre supplements. As the self‐selected sample size was limited, future research should examine the use of behavioural AI tools and personality using larger and longitudinal samples.

**What is already known about this subject**

- Experiences of referral through to programme delivery influence patients lived experience of the NHS England low calorie diet programme.
- NHS England low calorie diet programme service users mostly reported positive experiences with changes reported to diet and physical activity, and a perception that the programme was life changing.
- NHS England low calorie diet programme service users reported that a stronger person‐centred approach might further improve effectiveness and service user experience.

**What this study adds**

- This is the first study to examine the impact of personality on outcomes from the NHS England low calorie diet programme.
- This is the first study to use a unique Behavioural Artificial Intelligence tool to examine whether personality is associated with the lived experience of the low calorie diet pilot programme.
- This study provides impetus for exploring the use of behavioural AI within public health interventions and personalisation of approaches using personality.

## 1 INTRODUCTION

The prevalence of both obesity and type 2 diabetes (T2D) remains high across the world.^1^ In the UK, 25.9% of adults are living with obesity (UK Government, 2023)^2^ and 4.4 million live with diabetes (approximately 90% of whom have T2D).^3^ A result of this high prevalence is the increased risk of associated long‐term health conditions and the concomitant health and financial impact to the individual, health system, and wider society. Interventions to manage T2D and obesity range in type and intensity, with most requiring individual agency, focusing on reducing energy intake.

One such intervention that has shown some promise is total diet replacement and behaviour change approaches, with evidence from research such as the DiRECT (diabetes remission clinical trial) and DROPLET (doctor referral of overweight people to low energy to total diet replacement treatment) studies, that they can support weight loss, improve glycaemic control and remission of type 2 diabetes (HbA1c (average blood glucose levels over 2–3 months) <48 mmol/mol, without the use of glucose‐lowering medications).

In 2020, NHS England launched a low calorie diet (LCD) programme pilot based on the DiRECT study, recruiting across 10 initial (then a further 11 in 2022) sites across England.^4^ To be eligible for the programme, service users needed to have received a diagnosis of T2DM within the last 6 years, be non‐insulin dependent, and have a BMI ≥27 kg/m^2^ (or ≥25 kg/m^2^ for Black, Asian and minority ethnic communities). The programme consisted of three phases: total diet replacement (TDR) lasting 12 weeks, food reintroduction (13–18 weeks) and weight maintenance until the end of the programme (52 weeks). Different behaviour change delivery models were included in the pilot, including one‐to‐one, group, and digital. Cross‐sectional surveys were distributed via LCD site providers to service users to explore the lived experience of the programme.

A longstanding issue within T2DM treatment programmes is the impact of uptake and retention of people,^5^ warranting an improved understanding of the factors that influence uptake and retention. Tailoring or personalising approaches offers a potentially valuable approach that goes beyond demographic data to understand and utilise factors influencing motivation and ultimately behaviour. Artificial intelligence (AI) has received substantial focus over the last decade, offering an avenue for personalising healthcare.^6^ One such method is the use of behavioural AI that infers personality attributes as a means of predicting the experience of healthcare amongst people living with obesity; this research, examining the experiences reported by people living with obesity in NHS England, has highlighted that patient experiences differ based on personality attributes, values and sentiment.^7^

This study presents behavioural AI analysis of cross‐sectional survey data captured at the end of the 12‐week TDR phase (12 weeks). The NHS LCD pilot (as of June 2023, renamed the NHS type 2 diabetes path to remission programme) was a 1‐year programme consisting of 12 weeks of total diet replacement, 4–6‐week food reintroduction, followed by weight maintenance support,^8^ using a range of different behaviour change techniques.^9^ This analysis aimed to examine whether personality is associated with the lived experience of the LCD pilot programme.

## 2 MATERIALS AND METHODS

### 2.1 Design

The cross‐sectional surveys were co‐developed with NHS England, Diabetes UK, service providers, service users and the study Patient and Public Involvement and Engagement group. Surveys were completed anonymously online via Qualtrics (Provo, UT) software, and service users were invited to participate by an email sent to them by their service provider between September 2021 and April 2023. The content of the survey was presented in two parts: (1) experiences of the programme; (2) lifestyle, physical health, and wellbeing. This paper reports on data collected in the second part of the survey, in addition to linked sociodemographic data collected by NHS England (NHSE) as part of LCD programme monitoring. This study received ethical approval from the Health Research Authority (REF 21/WM/0136).

### 2.2 Service users

Of the 580 service users who started the 12‐week survey, 278 started and 184 completed the survey. Of the 184 surveys that were completed, 76 had a word count above the threshold of 100 words for scaled insights behavioural AI analysis and were included in the analysis presented in this study; see data analysis for description.

### 2.3 Measures

#### 2.3.1 Wellbeing

Emotional wellbeing was assessed using the short Warwick‐Edinburgh Mental Wellbeing Scale.^10^ The SWEMWS consists of seven items, each scored on a Likert scale ranging from 1 to 5. Items are summed and raw scores are then transformed into metric scores. Scores range from 7 to 35, with higher scores indicating better mental wellbeing. Previous research has reported good internal consistency, with Cronbach's alphas between .8 and .95.^10^

#### 2.3.2 Emotional eating

The six‐item emotional eating subscale of the Three Factor Eating Questionnaire was used (TFEQ).^11^ The scale uses a Likert scale ranging from 1 to 4 with higher scores indicating higher levels of emotional eating. The subscale has good internal consistency, with previous research reporting strong reliability for its use in measuring emotional eating in people living with obesity (i.e., Cronbach's alpha >.75).^12^

#### 2.3.3 Physical activity

To assess amount of physical activity, a single‐item physical activity tool was used asking respondents, “In the past week, on how many days have you done a total of 30 min or more of physical activity which was enough to raise your breathing rate.” This single‐item question was developed by and reported as performing as well as other short‐term physical activity tools for reliability and concurrent validity.^13^

#### 2.3.4 Pain

Joint pain was assessed using an 11‐point numerical rating scale, using the anchor question ‘Taking into account all of your joints, how would you rate your average pain over the last 7 days on a 0–10 scale, with 0 being ‘no pain’ and 10 being ‘pain as bad as it could be?’ Numerical rating scales are a unidimensional measure of pain intensity, recommended as a core outcome measure for assessing chronic pain due to conditions such as osteoarthritis.^14^ They have been found to be reliable and demonstrate good face and criterion validity.^15^, ^16^, ^17^

#### 2.3.5 Motivation and fibre supplement use

Several single‐item 5‐point Likert‐type scale questions were included within the survey relating to motivation to manage diabetes, weight, rating of the TDR products and frequency of fibre supplements*:

1. Currently, how motivated are you to manage your diabetes?
2. Currently, how motivated are you to lose weight or maintain your weight loss?
3. On average, how would you rate the TDR products you used?
4. On average, how often did you use the fibre supplements provided during the TDR phase?

#### 2.3.6 Open‐text questions

Several open‐ended questions were asked within the survey regarding patients experiences of the programme. Responses to these questions provided the language samples needed to infer personality features using the scaled insights behavioural AI tool (see Section 2.4 for description of the tool).

1. From referral up to today, is there anything that could have been done differently to improve your experience so far? Yes/no (where participants responded yes, they were asked to provide detail)
2. Why did you decide to take part in the programme?
3. What would success on the programme look like for you?
4. Is there anything you would change about the programme that would have improved your experience so far?
5. Is there anything else you would like to tell us about (e.g., significant life events, religious or cultural circumstances) that have affected your experience on the programme so far?

### 2.4 Data analysis

Where service users had amassed 100 or more words within open‐ended responses (*N* = 76), these data were analysed using the scaled insights behavioural AI tool. The scaled insights behavioural AI tool takes as input a language sample and produces 113 personality features.^7^ Participant responses to the open‐ended questions amassed between 251 and 1939 words, with a mean of 337.85 (SD—96.69) words. Following this, features were used as input into the multiple machine learning models, which were used in two settings: unsupervised (clustering) and supervised (classification).^7^ We investigated to what extent features obtained from a language sample are correlated with emotional wellbeing, physical activity, pain, motivation to manage diabetes, motivation to lose weight, rating of TDR products and frequency of using fibre supplements. We then conducted one‐way analysis of variance to examine differences between the three clusters based on emotional wellbeing, physical activity, pain, motivation to manage diabetes, motivation to lose weight, rating of TDR products and frequency of using fibre supplements. The scaled insights behavioural AI tool utilises Python's library scikit‐learn to train and test each of the 113 machine learning models personality traits. The Python libraries scipy, numpy, sklearn were used to perform the statistical analysis, construct personality clusters, and generate the scatter plot. For all statistical tests, alpha was .05.

Scaled insights behavioural AI tool meticulously analyses textual data, involving written content, as a means of deriving intricate personality insights. By using sophisticated natural language processing (NLP) techniques, the tool delves into linguistic cues, dissects writing style nuances and scrutinises content details, extracting a comprehensive set of features corresponding to 113 personality features, combining different models such as Big Five and Human Values.^17^ This in‐depth analysis encompasses the examination of word choices, the structure of sentences and the nuanced emotional tones embedded within the text, culminating in the creation of an individualised personality profile. These personality profiles provide invaluable information about a broad spectrum of traits, including but not limited to openness, extraversion, conscientiousness, drives, needs, values, thinking styles and sentiments. These insights, meticulously gleaned through the scaled insights' behavioural AI, enable professionals to facilitate and support patients in adhering to health‐promoting behaviours or making well‐informed decisions, leveraging the power of effective nudges and personalised communication. The scaled insights' behavioural AI tool extends its utility beyond mere profiling. It can be used to construct predictive models that forecast how an individual's personality is likely to influence their behaviours and subsequent outcomes, providing a holistic understanding that can empower professionals to optimise their interventions and support strategies (see Figure S1 and Table S1).

## 3 RESULTS

Based on scaled insights behavioural AI analysis, there were three personality clusters with distinct profiles: Cluster 1 (“Disciplined Achievers”) characterised by high conscientiousness, self‐discipline, and work orientation. This group is more structured and focused on responsibilities; Cluster 2 (“Emotionally Reactive”) characterised by high anxiety, neuroticism and vulnerability. This cluster tends to struggle with emotional stability and structure; and Cluster 3 (“Balanced Organisers”) characterised by moderate conscientiousness, vulnerability and higher orderliness. Personality clusters are constructed based on the most differentiating personality features (i.e., the traits that load onto the personality clusters; see Table 1).

**TABLE 1:** Cluster centroids for the 10 features with the greatest absolute value differences between clusters.

Feature	Disciplined achievers	Emotionally reactive	Balanced organisers
Anxiety	0.31 (0.16)	0.62 (0.14)	0.28 (0.13)
Work oriented	0.61 (0.12)	0.51 (0.13)	0.27 (0.19)
Self‐discipline	0.52 (0.20)	0.17 (0.11)	0.41 (0.11)
Neuroticism	0.45 (0.24)	0.78 (0.14)	0.52 (0.28)
Vulnerability	0.47 (0.15)	0.77 (0.09)	0.48 (0.15)
Orderliness	0.40 (0.21)	0.17 (0.10)	0.49 (0.19)
Conscientiousness	0.47 (0.16)	0.21 (0.10)	0.49 (0.18)
Self‐efficacy	0.42 (0.17)	0.24 (0.15)	0.14 (0.09)
Dutiful	0.62 (0.12)	0.37 (0.10)	0.40 (0.12)
Activity level	0.48 (0.13)	0.30 (0.15)	0.21 (0.10)

*Note*: All scores are within (0, 1).

Abbreviations: *p*, *p* value; SD, standard deviation.

Service users in Cluster 1, the “Disciplined Achievers” scored higher for work orientation, self‐discipline, and conscientiousness compared to Clusters 2 and 3 (see Table 1). Cluster 2 respondents, the “Emotionally Reactive” are more prone to being emotional and neurotic. Cluster 3, the “Balanced Organisers,” is more ordered and calmer in its approach. Table 1 presents the top 10 personality features that differentiate the three clusters. A significant main effect was identified for each of the personality traits presented in Table 1.

*Anxiety*. Post‐hoc tests identified significant differences in anxiety between the Emotionally Reactive and Balanced Organisers (*F*(2,67) = 22.79, *p* < .001) and the Disciplined Achievers and Balanced Organisers (*F*(2,67) = 22.79, *p* < .05) personality clusters. There was no significant difference between the Disciplined Achievers and Emotionally Reactive (*F*(2,67) = 22.79, *p* > .05) personality clusters.

*Self‐discipline*. Post‐hoc tests revealed a significant difference in self‐discipline between the Emotionally Reactive and Balanced Organisers (*F*(2,67) = 29.38, *p* < .001). There were no significant differences between the Disciplined Achievers and Emotionally Reactive and Disciplined Achievers (*F*(2,67) = 29.38, *p* > .05) and Balanced Organisers (*F*(2,67) = 29.38, *p* > .05) personality clusters.

*Neuroticism*. Significant post‐hoc tests were identified for neuroticism between the Emotionally Reactive and Balanced Organisers and Disciplined Achievers (*F*(2,67) = 21.02, *p* < .001) and Balanced Organisers (*F*(2,67) = 21.02, *p* < .05) personality clusters. There was no significant difference between the Disciplined Achievers and Emotionally Reactive personality clusters (*F*(2,67) = 21.02, *p* > .05).

*Vulnerability*. Post‐hoc tests identified significant differences in vulnerability between the Emotionally Reactive and Balanced Organisers and Disciplined Achievers (*F*(2,67) = 20.85, *p* < .001) and Balanced Organisers (*F*(2,67) = 20.85, *p* < .05) personality clusters. There was no significant difference between the Disciplined Achievers and Emotionally Reactive personality clusters (*F*(2,67) = 20.85, *p* > .05).

*Orderliness*. Post‐hoc tests revealed a significant difference in orderliness between the Emotionally Reactive and Balanced Organisers (*F*(2,67) = 19.44, *p* < .001) and Disciplined Achievers and Balanced Organisers (*F*(2,67) = 19.44, *p* < .05) personality clusters. There was no significant difference between the Disciplined Achievers and Emotionally Reactive (*F*(2,67) = 19.44, *p* > .05) personality clusters.

*Conscientiousness*. Significant post‐hoc tests were identified for conscientiousness between the Disciplined Achievers and Emotionally Reactive (*F*(2,67) = 44.95, *p* < .001) and the Emotionally Reactive and Balanced Organisers (*F*(2,67) = 44.95, *p* < .001) personality clusters. There was no significant difference between the Disciplined Achievers and Balanced Organisers (*F*(2,67) = 44.95, *p* > .05) personality clusters.

*Dutiful*. Post‐hoc tests revealed a significant difference in dutifulness between the Emotionally Reactive and Balanced Organisers (*F*(2,67) = 18.53, *p* < .001) and Disciplined Achievers and Balanced Organisers (*F*(2,67) = 18.53, *p* < .05) personality clusters. There was no significant difference between the Disciplined Achievers and Emotionally Reactive (*F*(2,67) = 18.53, *p* > .05) personality clusters.

Post hoc tests revealed no significant differences between the personality clusters for the traits work‐oriented, self‐efficacy, and activity level (*p* > .05).

**FIGURE 1:**
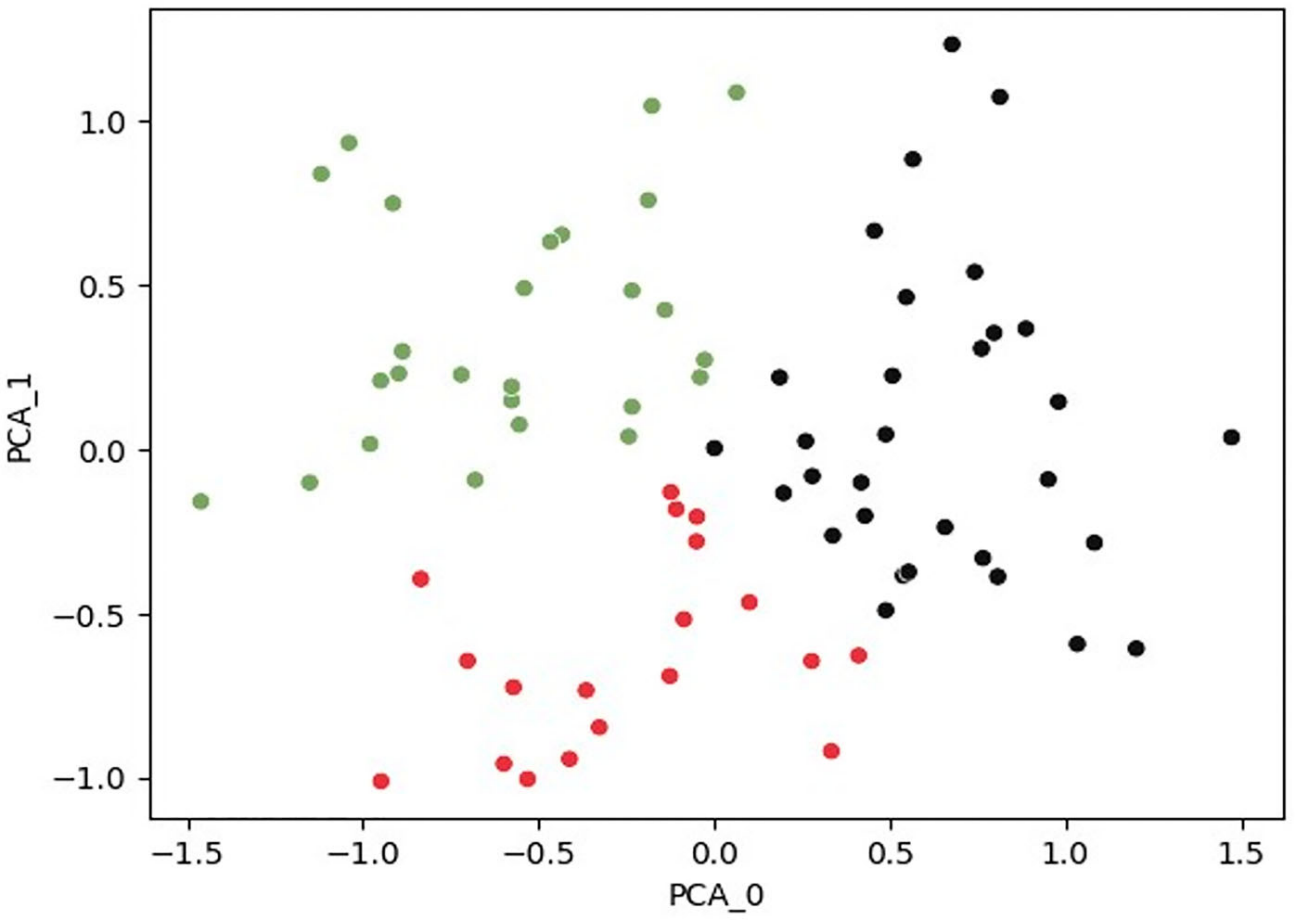
Personality cluster distribution using scaled insights behavioural AI tool.

Scaled insights behavioural AI analysis of personality features demonstrates that there are three distinct personality clusters within this sample of respondents to the LCD survey (Figure 1).

Table 2 reports the mean scores for wellbeing, motivation to manage diabetes, physical activity, pain, emotional eating, rating of diet replacement supplements, frequency of using fibre supplements and motivation to lose weight for each of the three clusters and the *p* value for comparing the three clusters based on these measures. In all instances, there was no significant difference based on personality cluster for wellbeing, motivation to manage diabetes, physical activity, pain, emotional eating, rating of diet replacement supplements, frequency of using fibre supplements and motivation to lose weight (*p* > .05).

**TABLE 2:** Mean, standard deviation for wellbeing, motivation to manage diabetes, physical activity, pain, emotional eating, rating of diet replacement supplements, frequency of using fibre supplements and motivation to lose weight by personality cluster.

Cluster	*N*	Motivation to manage diabetes	Motivation to lose weight	Rating of TDR products	Frequency of fibre supplements	SWEMWS score	Pain score	Emotional eating	Physical activity
Disciplined Achievers	27	4.92 (0.27)	4.88 (0.32)	3.85 (0.82)	3.73 (1.51)	31.38 (3.17)	2.69 (1.92)	8.96 (3.03)	3.81 (2.37)
Emotionally Reactive	31	4.29 (0.59)	4.43 (0.50)	3.50 (1.18)	3.79 (1.65)	23.50 (5.29)	8.00 (1.65)	12.43 (3.64)	2.57 (2.35)
Balanced Organisers	18	4.69 (0.52)	4.92 (0.28)	3.83 (0.96)	3.72 (1.57)	27.92 (3.23)	3.56 (2.35)	18.28 (3.28)	2.64 (2.19)
Range	‐	1–5	1–5	1–5	1–5	5–35	0–10	4–24	0–7
Context	Total N = 76	5: Very motivated, 1: Not at all motivate	5: Very motivated, 1: Not at all motivated	5: Very nice, 1: Horrible	5: Twice a day, everyday. 1: Never or very rarely	Low: poor wellness, high: high wellness	0: low pain, 10: high pain	Low: emotional eater, high: emotions do not influence eating	Number of days in past week where 30 min workout was done
*p* value	‐	0.19	0.87	0.41	0.48	0.05	0.23	0.38	0.21

Abbreviations: *N*, sample size; SWEMWS, short Warwick‐Edinburgh Mental Wellbeing Scale; TDR, total diet replacement.

## 4 DISCUSSION

This study aimed to explore whether personality is associated with the lived experience of the LCD pilot programme. The scaled insights behavioural AI tool identified three distinct personality clusters that differed significantly based on personality traits that loaded onto them. As such, the first cluster, “Disciplined Achievers” is characterised by high conscientiousness, self‐discipline and a strong work orientation. Service users in the second cluster, “Emotionally Reactive” had high anxiety, neuroticism and vulnerability, and in the third cluster, “Balanced Organisers” service users had moderate conscientiousness, vulnerability and high orderliness. They balance emotional control and organisation better than Cluster 2 but are not as disciplined as Cluster 1. Descriptive findings indicate that service users across all three personality clusters reported high motivation to manage diabetes and to lose weight. Previous research has highlighted that motivation to manage diabetes and to lose weight often dissipates by week 12, particularly in community‐based weight management programmes, and is suggested as a key reason for the high attrition rates reported.^18^ The high motivation may represent the clinical support provided to service users within the programme and potentially in response to reported significant weight reduction similar to clinical trials.^8^ Thus, further exploration that teases out the key factors that influenced motivation during the programme is warranted, as well as other factors that influence behaviour change, e.g., biological, societal and environmental factors.

Overall, none of the survey outcome measures were significantly different between the three personality clusters. Previous research has reported that personality affects wellbeing within interventions or in response to public health emergencies/threats,^19^ and thus, the non‐significance of the findings may have been influenced by the relatively small sample size. Further research that recruits a greater sample may therefore tease out whether personality does affect the lived experience of the LCD programme.

Descriptive findings do suggest that personality may play a role in patients lived experience of the LCD programme, where, for instance, service users in Cluster 1 reported higher wellbeing, higher physical activity, lower pain, and lower emotional eating compared to Clusters 2 and 3, whilst Cluster 2 reported the lowest wellbeing, highest pain and emotional eating. Service users across the three personality clusters reported high motivation to manage diabetes and to lose weight and thus may not be representative of all service users who participated in the LCD programme. However, as highlighted elsewhere in evaluating the LCD programme,^20^ the majority of people were motivated and indeed were recruited based on a high motivation both to manage diabetes and lose weight.

Research from the LCD programme reported that wellbeing was related to emotional eating.^21^ Marwood et al.^21^ suggested that the presence of a potential binge eating disorder diagnosis was demonstrated in 24.3% of the sample. Importantly, they also suggested that being female and engaging in more frequent weight cycling was associated with higher emotional eating and a greater likelihood of binge eating.

This study is not without its limitations. First, the low numbers of service users (*N* = 76) providing sufficient words in response to open‐ended survey questions limited the sample size, and recruitment would have allowed for stronger comparison of the distinct personality clusters and greater confidence in the results presented here. Second, we present cross‐sectional analysis as we had insufficient completion within the baseline, 18‐ and 52‐week surveys to facilitate a longitudinal analysis over the course of the LCD programme. This would have provided the opportunity to examine whether personality is associated with greater adherence and outcomes from participating in the LCD programme. Future work to examine the impact of the LCD programme and other longer‐term interventions should consider mechanisms to improve uptake and repeated participation. Third, the nature of the study meant we needed to take a pragmatist approach to creating the survey. As such, we created bespoke questions to assess motivation to manage type 2 diabetes and to lose weight rather than utilising validated measures. Fourth, the measurement of motivation in this study reflects intention and not actual behavioural change. Further study is therefore suggested that explores the association between personality and behaviour change related to the management diabetes and weight may require further exploration. Finally, we did not have access to data relating to the participants service level data (i.e., weight, programme retention or engagement) and therefore were unable to explore associations between personality and these data. Previous research has highlighted that personality is a factor influencing adherence‐related behaviours including weight management and dietary.^22^, ^23^

To our knowledge, this is the first study to explore the impact of personality in predicting behaviours and outcomes associated with LCD programmes, and in doing so, presents several areas for future research. Given that this study has demonstrated the feasibility of clustering participants based on personality even with the limited sample size, future research should explore whether personality is associated with the lived experience, adherence and outcomes of type 2 diabetes and obesity treatment programmes. Other research such as the English NHS diabetes prevention programme has likewise shown that psychosocial factors may offer greater insights and allow for improved approaches that benefit uptake and adherence beyond fixed factors such as demographics.^24^ Our intention was to examine whether participants' experience of the programme differed across the timepoints of the LCD programme, and whether personality was a key determining factor. Only a small amount of LCD programme participants completed the survey at one time point and thus, longitudinal analysis was not possible. Future research should explore longitudinal differences in participants' experiences of LCD programmes based on personality.

Use of AI within public and clinical health to provide unique insights that advance care has become a major focus in the UK and globally.^25^ Whilst this pilot study has some limitations, it also highlights the potential use and novelty of understanding patient engagement, experience and outcomes. This pilot study has demonstrated that there is the potential of a unique behavioural AI tool to understand factors that influence uptake and adherence to public health interventions such as the LCD programme; and there is an impetus for larger scale work that teases out the importance and potential implications of personality and AI within public health interventions. As such, AI may present a tool that can support interventions such as LCD programmes through improved approaches to patient care and the development of more tailored, personalised approaches that go beyond typical demographic segmentation as where patients and the public are profiled.

## FUNDING INFORMATION

This work was supported by the National Institute for Health Research, Health Services and Delivery Research [NIHR 132075]. The NHS LCD programme is funded by NHS England. The views expressed in this publication are those of the author(s) and not necessarily those of the MRC, NIHR or the Department of Health and Social Care.

## CONFLICT OF INTEREST STATEMENT

SWF declares researcher‐led grants from the National Institute for Health Research, the Office of Health Improvement and Disparities, Doncaster Council, the West Yorkshire Combined Authority and Novo Nordisk; and support for attending academic conferences from Johnson & Johnson, Novo Nordisk, Devon NHS Integrated Care Service, the UK Parliament and Safefood. SWF also declares employment at Scaled Insights. EG declares employment at Scaled Insights. MK declares employment at Scaled Insights. SS declares employment at Scaled Insights. DR declares funding from the National Institute for Health Research and the Welsh Government. SRK declares supported in part by the National Institute for Health Research Leeds Biomedical Research Centre. LE declares funding from the National Institute for Health Research, the Medical Research Council, and the Nuffield Foundation; she is also an independent member of the HOT/COT steering group and ACTION teams project but receives no funding for this.

## Supporting information


**FIGURE S1:** Scaled Insights proprietary tool data flow architecture.
**TABLE S1:** Personality profile characteristics derived from Scaled Insights Behavioural AI tool.

## Data Availability

The data that support the findings of this study are available from the corresponding author upon reasonable request.

## Footnotes

* Fibre supplement use was asked because NHSE service specification informs that providers need to provide fibre supplements during the TDR phase where required by the service user.
