# Supplementary material for: Is personality associated with the lived experience of the NHS England low calorie diet programme: A pilot study
Source: Clin Obes. 2025 Feb 25;15(4):e70003. doi: 10.1111/cob.70003 (PMC12289402; doi:10.1111/cob.70003)
Supplement: Supplementary file 1 — FIGURE S1: Scaled Insights proprietary tool data flow architecture. TABLE S1: Personality profile characteristics derived from Scaled Insights Behavioural AI tool. [file COB-15-e70003-s001.docx]

**Supplemental Material**

**Figure S1.** Scaled Insights proprietary tool data flow architecture

*
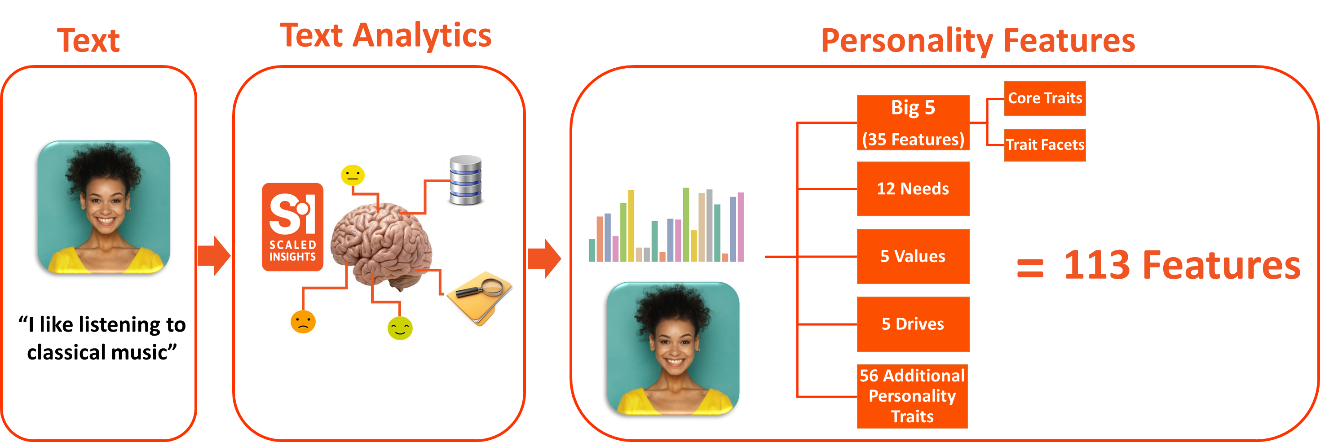
*

**Table S1.** Personality profile characteristics derived from Scaled Insights Behavioural AI tool

| **Feature** | **Description** |
| --- | --- |
| Emotionality | Aware of your feelings and how to express them. |
| Modesty | Uncomfortable being the centre of attention. |
| Social Group Orientated | Measures the degree to which a person’s values and behaviours are rooted in their sense of family. |
| Distractible | Feel your desires strongly and are easily tempted by them. |
| Self-Disciplined | Can tackle and stick with tough tasks. |
| Openness | The extent to which a person is open to experiencing different activities. |
| Anxiety | Tend to worry about things that might happen. |
| Vulnerability | Easily overwhelmed in stressful situations. |
| Workhorse | Measures the degree to which a person has a strong work ethic vs. preference for leisure and non-work activity. |
| Grounded | Exhibits groundedness and a desire to hold things together. They need things to be well organised and under control. |
| Happiness | Measures the degree to which a person is optimistic, upbeat, and happy. |
| Neuroticism | Measures the degree to which a person expresses strong negative emotions. |
| Aggression | Measures the degree to which a person exhibits anger or aggression. |
| Cold | Measures the degree to which a person is emotionally unresponsive and has difficulty empathising with others. |
| Depression | Measures the degree to which a person may have difficulty finding joy in their life. |
| Body Focus | Measures the degree to which a person focuses attention on their body or other people's bodies. |
| Persuasive | Measures the degree to which a person is able to create rapport with the intention of persuading others. |
| Extraversion | Person's tendency to seek stimulation in the company of others. |
| Sensory Processing Sensitivity | The extent to which a person's emotions are sensitive to the person's environment. |
| Conscientiousness | Person's tendency to act in an organised or thoughtful way. |
| Agreeableness | Person's tendency to be compassionate and cooperative toward others. |
